# Performance control during longitudinal activation fMRI studies

**DOI:** 10.3389/fnhum.2024.1459140

**Published:** 2024-10-15

**Authors:** Martin Lotze

**Affiliations:** Functional Imaging Unit, Institute for Diagnostic Radiology and Neuroradiology, University Medicine of Greifswald, Greifswald, Germany

**Keywords:** fMRI, performance control, sensorimotor, cognition, emotional processing, sensory modality, data evaluation, psychophysiology

## Abstract

The documentation of performance during functional imaging represents a standard procedure employed to control for compliance, sensorimotor, and cognitive demands. In the case of motor tasks, preciseness, force, and frequency have a significant impact on the magnitude of functional activation. Questionnaires are used in psychological investigations to control for cognitive demand, while psychophysiological documentation is employed to record bodily responses. For longitudinal intervention studies, it is of utmost importance to implement meticulous pre- and post-performance controls and balance to accurately assess changes over time. Any changes in compliance may introduce additional uncontrolled variables, which can hinder the interpretation of functional magnetic resonance imaging (fMRI)-related changes. This narrative review presents strategies for controlling and balancing performance in functional imaging approaches to document neuroplasticity in rehabilitative studies. These strategies include not only motor-related aspects, such as precision, velocity, and force, but also timing aspects, such as the start and stop of movement periods. In addition, it discusses strategies for the modulation and control of movement aspects with visual feedback, as well as for the control of physiological changes during experimental modulation. Although these measures require additional care, which is often more demanding than the neuroimaging part of the study, they are crucial for a relevant interpretation and publication of fMRI studies.

## Introduction

Longitudinal studies are challenging due to the extensive duration required, the need for patient compliance, and the balancing of patients’ performance during pre- and post-measurements. However, these studies offer multiple additional possibilities for evaluating disease progression or changes in neural substrates for specific tasks during interventions. In addition, longitudinal studies are necessary to identify predictive biomarkers for treatment response. Neuroimaging and neurophysiology can provide substantial data in such treatment studies, especially if the underlying neural mechanisms of behavioral changes are unclear. For diseases with high variability between patients (e.g., stroke or chronic pain), pre–post designs enhance statistical power and add value to the data. Here, interindividual baseline differences are less important when changes over interventions are the focus of interest, allowing for pre–post comparisons on an individual level. Correlation analyses can then identify the areas where fMRI activation covaries along with changes in outcomes. A short overview of the evaluation of longitudinal fMRI data has been provided by Skup (2010). Performance data can be incorporated into different statistical models, for instance, using regression analyses. Typical findings would, therefore, include identifying those brain areas that are associated with recovered upper limb rehabilitation following a stroke (e.g., Horn et al., 2016). In this review, we focus exclusively on task-related fMRI (in the following fMRI) and do not cover resting state fMRI.

In contrast to structural MRI, task-related fMRI depends on the time dimension, and problems related to the onset, duration, and intensity of performance significantly modify both local (amplitude) and dynamic (connectivity strength) imaging parameters. Changes in activation magnitude over time, and group differences, might well be based on performance differences during the functional imaging task. It is crucial to control for compliance with the protocol to exclude outliers during measurement. Performance control should be easily accessible, storable in a widespread format, and capable of enabling a meaningful quantification of the relevant aspects of the task. Box 1 provides criteria to be solved by performance control.

Box 1 **Overview on criteria for performance control.**

For all fMRI-measurements:

(1) Is the participant awake and follows the instructions (response time, vigilance rating, and accuracy of responses)?

(2) How does he/she rate the stimulus (e.g., emotional ratings for valence and arousal)?

(3) How does he/she feel during the experiment (concentration, comfort, cognitive or emotional distress)?

(4) Can we integrate psychophysiological data (SCR, startle, heart rate, respiration)?

For longitudinal assessments:

(1) Does the patient do the same during the post-measurement as during the baseline measurement?

(2) Can we balance the base line and post-measurements performance?

This article provides a narrative overview of solutions for controlling motor performance in different fMRI-paradigms, which can also be applied for longitudinal interventions in neurorehabilitation studies. This study outlines our solutions for controlling motor performance in neurorehabilitative trials, differentiating them into three categories: (1) research topics (processing of cognitive, emotional, sensorimotor, auditory, and visual trials), (2) types of performance control (e.g., sensorimotor, ratings, and psychophysiology), and (3) specific requirements for longitudinal approaches (e.g., balancing performance between time units).

### A more detailed review of the sensorimotor system

Depending on the task tested, we could differentiate between sensorimotor tasks, cognitive tasks, emotional processing trials, and social interaction experiments. To illustrate the importance of performance control and its effect on functional activation, the sensorimotor system might be best suited. There is a substantial body of literature on the modulation of fMRI activation in primary and secondary motor areas through force (e.g., Cramer et al., 2002; Dettmers et al., 1995) and frequency (e.g., Schlaug et al., 1996; Riecker et al., 2003).

**Figure 1:**
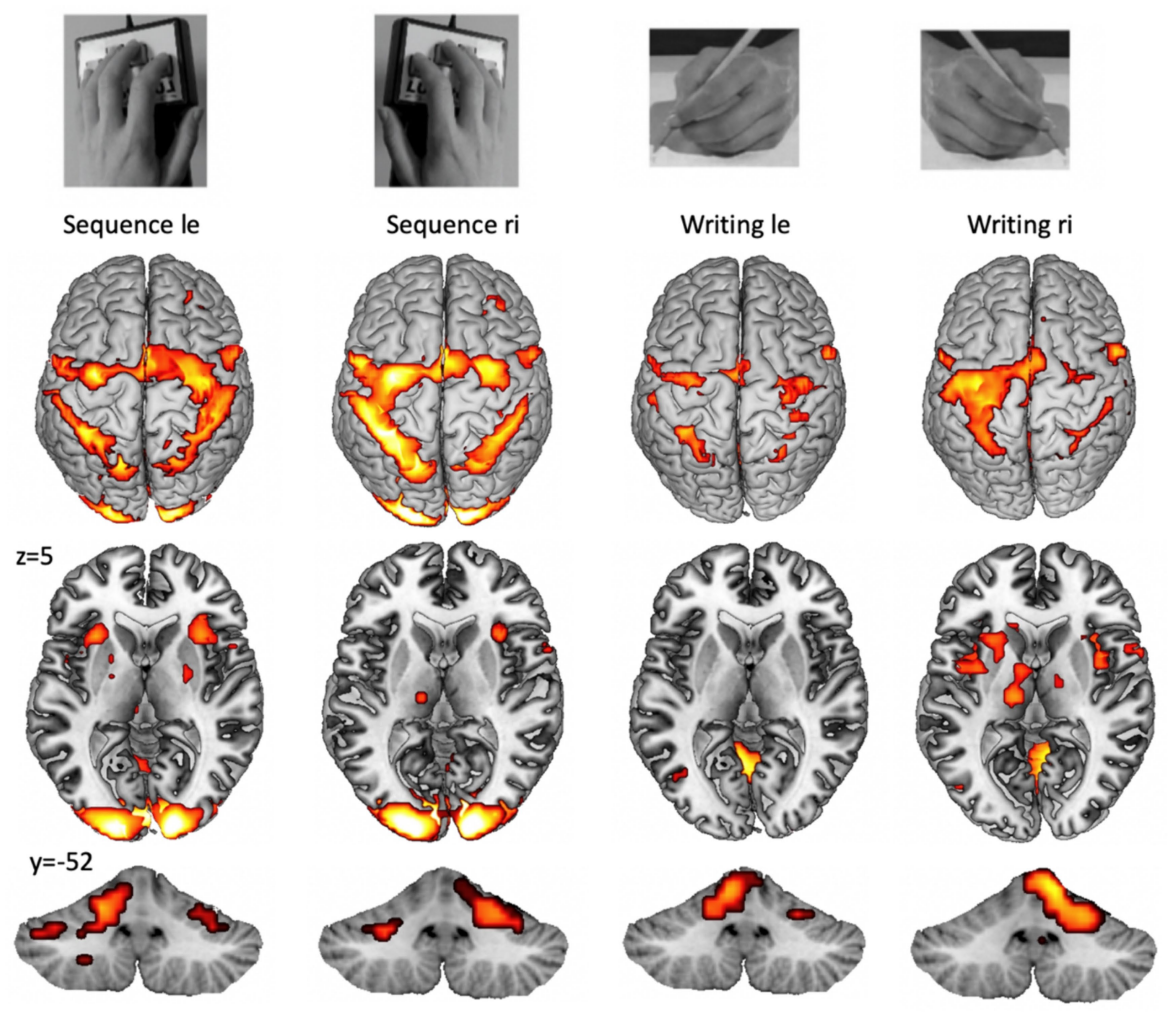
Example for cortical (top), subcortical (middle), and cerebellar (bottom) fMRI patterns during the performance of a unilateral finger sequence task and a unilateral writing task (copying words on a paper). Group activation with p_FWE_ < 0.05 from 15 participants (from the study: Walz et al., 2015). A highly trained task, such as writing with the dominant (right) hand (right part), shows higher lateralization and increased basal ganglia activation. Cortical lateralization is mirrored for the cerebellar hemispheres.

It has to be mentioned that other issues, such as the complexity or preciseness of task performance, also modify fMRI activation. An example of functional representations of complex unilateral upper limb tasks is provided in Figure 1.

For psychological trials, including emotional processing, social interaction, and cognitive tasks, we aim to obtain both ratings and psychophysical responses (skin conductance response (SCR), heart rate modulation, and electromyographic (EMG) measurements (e.g., startle responses)). These data can then be embedded in the functional imaging analysis for each stimulus separately (Anders et al., 2004) or averaged across each participant (Lebon et al., 2018). In the following section, we provide an overview of the methods important for embedding performance tests in an fMRI design and evaluate the data.

### Experimental planning for longitudinal designs

**Figure 2:**
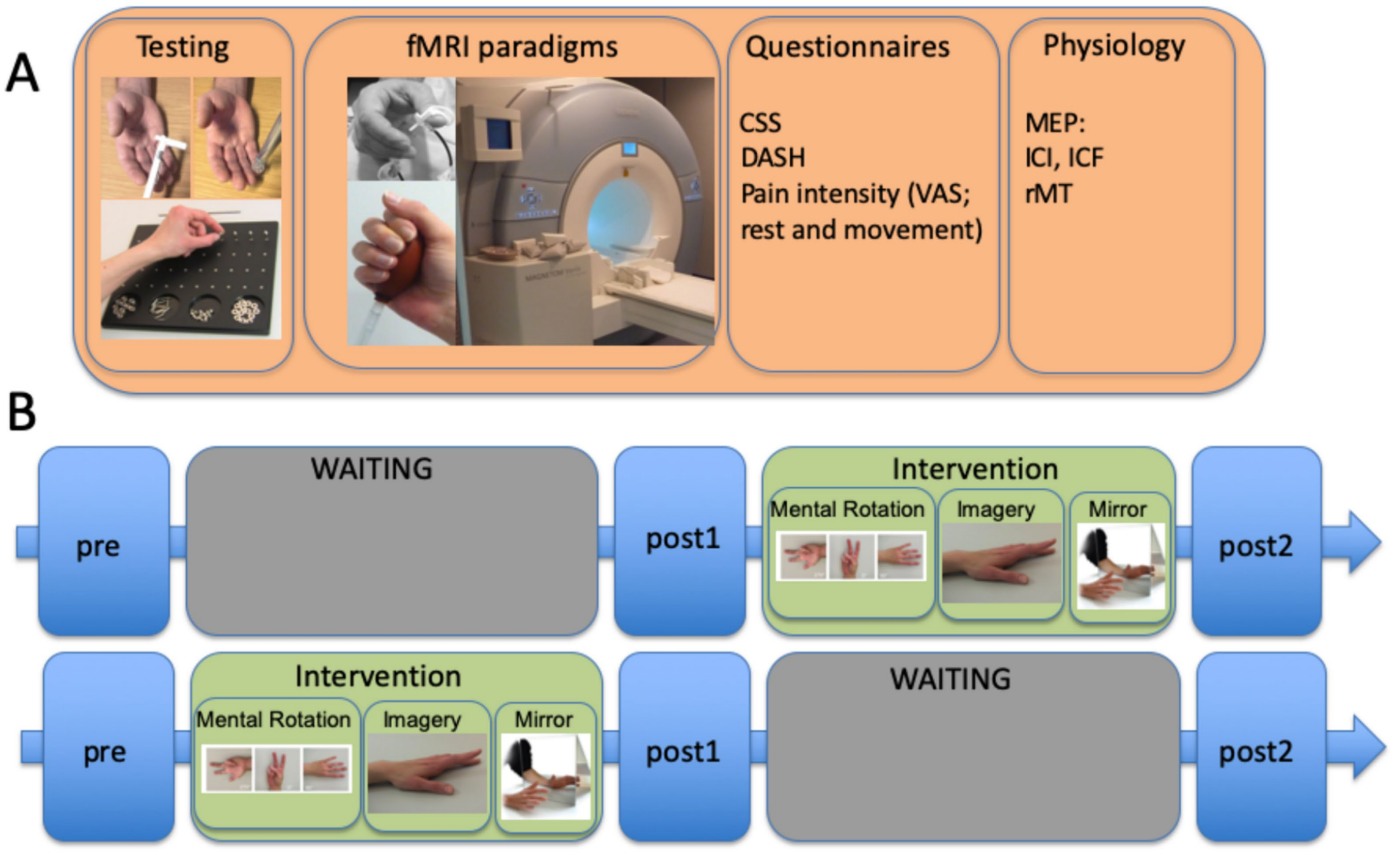
Example of a waiting list control study involving patients with complex regional pain syndrome (CRPS), including the time units for the INTERVENTION (graded motor imagery over 6 weeks) and WAITING periods with three different evaluation times. (A) measurements performed at each of the three time points included sensorimotor testing (von Frey hair test, spatiotactile resolution, and pinch grip performance/Roeder test), fMRI paradigms (somatosensory and motor), questionnaires CRPS severity score (CSS), DASH, and pain intensity using a visual analog scale (VAS), and physiology assessments [here, separately performed TMS measures testing motor evoked potentials (MEPs) for estimating intracortical facilitation (ICF), intracortical inhibition (ICI), and resting motor thresholds]. Somatosensory stimulation was coupled with a cognitive task to decrease habituation to the stimuli. (B) time course of the complete protocol, including the GMI intervention: 2 weeks of mental rotation were followed by 2 weeks of motor imagery, and then 2 weeks of mirror therapy. The training comprised at least eight sessions per day. The complete study is published by Strauss et al. (2021).

Figure 2 demonstrates a typical setup for a waiting list-controlled intervention. This design allows to control for the effect of an effectively proven intervention for patients in a chronic stable stage of disease; however, it is not suitable for patients in an unstable acute stage of disease.

Testing interventions during the acute stage usually do not allow for a waiting list control design because the onset of treatment is highly relevant. Most studies testing evidence-based interventions usually aim to test the underlying neural modulations of an effective intervention. When overseeing the complexity of an interventional study integrating fMRI, it becomes clear that these studies usually cannot be performed without substantial financial support.

### Integrating performance control in longitudinal fMRI studies

Planning the experimental setup and integrating performance tests can be quite demanding because of the delay in the BOLD response curve and the interaction of responses both within and between brain areas. Most fMRI designs include a baseline as a rest period and present different events either in a block (block design) or as a single event (event-related design). A mixed design combines the presentation of blocked and single events. The design is dependent not only on the task but also on the aim of the study: most motor tasks can be robustly performed in a block design (e.g., Lotze et al., 2012), whereas most stimulations [for the auditory domain and highly arousing stimuli, see Witt et al. (2023)] tend to habituate and are typically presented as a single event. With respect to study aims, a block design might not enable differentiation of functional representation in relation to a single event, whereas an event-related design with a given onset and duration enables a precise analysis of the timing of functional activation in relation to task performance or the stimulation protocol (e.g., Mihai et al., 2014). For patients with limited compliance (e.g., stroke), a block design is easier to implement and typically shows higher effect sizes for the BOLD amplitude in cortical areas compared to event-related designs.

Several reviews describe different fMRI designs and can help to optimize the paradigm (e.g., Maus and van Breukelen, 2013). If a baseline period is included, which is the case in most experimental setups, a BOLD-response curve delay of 6 s has to be considered. Usually, it takes approximately 10 s for the BOLD effect to return to baseline (Pollmann et al., 2000). In addition, depending on the duration of the stimulation/active period, a general guideline is to use baseline periods not shorter than 10 s. This is also important for any ratings performed by the participant during scanning, which increases the measurement time relevantly. If the investigation of an interaction of different stimuli is the primary aim of the study, an overlay of hemodynamic response curves is acceptable and could even improve comparisons between conditions (Pollmann et al., 2000). In addition, jittering of the stimulus onset might be necessary for valid behavioral responses; the participant cannot anticipate the onset time, thereby enabling reaction time measurements. Habituation and exhaustion are important factors that decrease statistical power and usually necessitate adjustments in the total measurement time and number of stimuli. It is important to consider that some psychophysiological variables need larger stimulus durations, e.g., skin conductance measurements require at least 6 s to develop a relevant response, which increases the total measurement time. Scanner-specific parameters (such as gradient, field strength, imaging sequence, repetition time, and shimming methods), the region of interest (high or low artifacts), and several other factors (e.g., multiband measurements) have a significant impact on the planning of the design of an experiment (Herrnberger, 2022). Therefore, fMRI designs represent a compromise between maximizing the effect size for the dependent variable (fMRI activation is optimal for a high number of events and a high number of scans per event), relevant response in psychophysiological measurements, habituation and tiredness effects, time necessary for preparing the participant and additional performance control devices in the scanner, available measurement time, and participant compliance.

### Embedding the participant’s response into the experimental design

**Figure 3:**
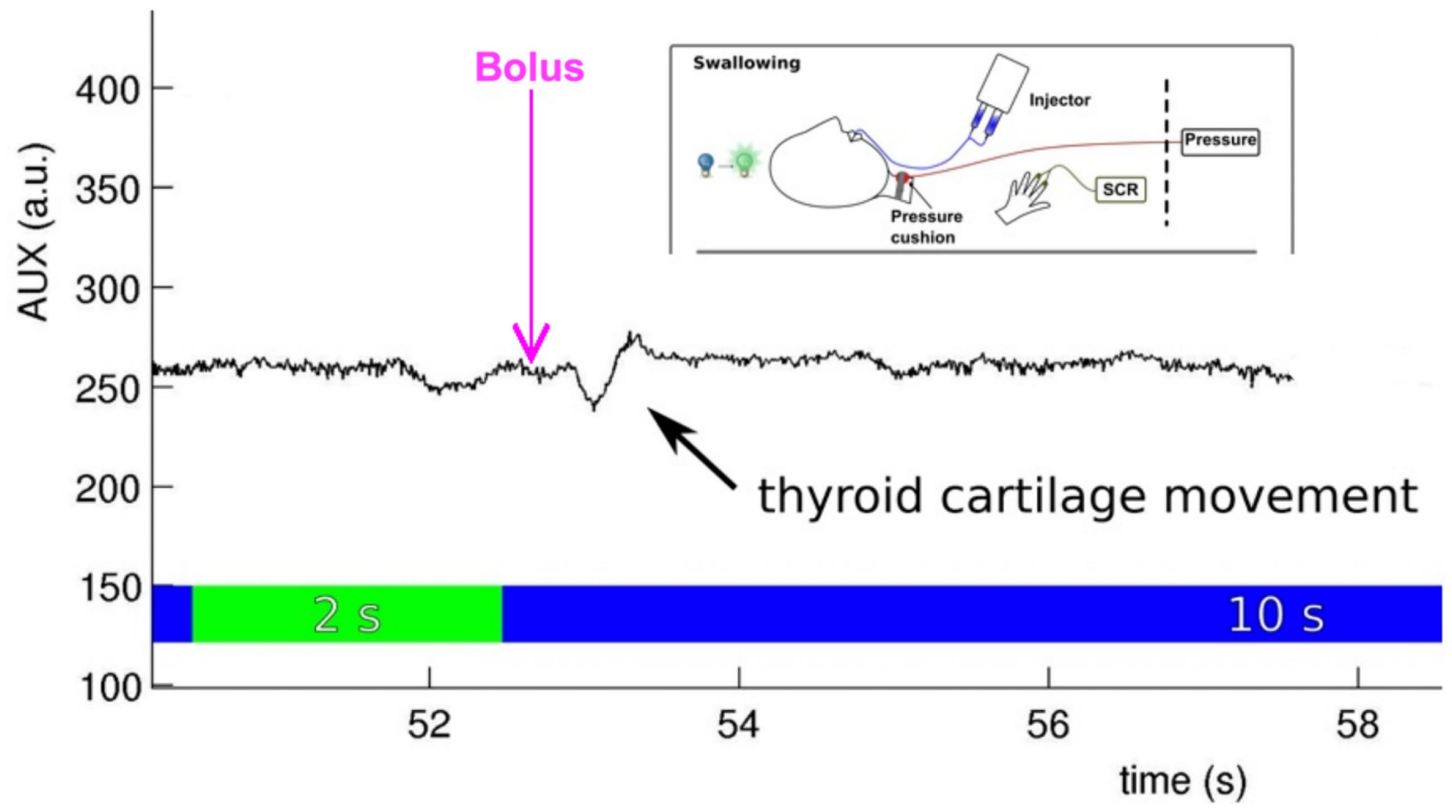
Embedding the stimulus onset (water bolus provided by an injector), psychophysiological measurement [skin conductance response (SCR) at the volar hand], and performance control (cartilage movement assessed with a pressure cushion) in an fMRI experiment testing effects during event-related swallowing [see Mihai et al. (2016)].

Along with a scanner-specific trigger box, the software used for presenting the experimental design also integrates the onset and duration of MRI measurements into the overall experimental protocol. In addition, it can precisely document the participant’s response button presses. It is important to consider that ratings not only add additional movement artifacts but also additional brain activation, which must be acknowledged for the timing of events and baseline in the fMRI study design (see above). For presenting fMRI paradigms, receiving trigger pulses from the MRI scanner, or storing participants’ performance, three software solutions are mainly used (James et al., 2014). These include Cogent, an open-source MATLAB toolbox; E-prime (especially E-Studio helps for creating experiments); and Presentation (Neurobehavioral Systems, Inc., California, United States). All these software solutions interact with the scanner (the MRI triggers the presentation software), activate the stimulation device, integrate feedback from participants (e.g., assessments of emotional pictures), and store log files for documentation. Psychophysiological data are usually recorded using external software (e.g., BrainVision Analyzer 2.0; Brain Products, Gilching, Germany) and interact with the stimulus Presentation software, providing onset markers. An example of a setup that includes different psychophysiological recordings in a neurorehabilitative study involving dysphagia patients is shown in Figure 3.

### General assessments and scoring

Scoring of mood or cognition is usually performed before and/or after the MRI session. The easiest way to add the actual scores of participants is to ask for them between measurement slots, even though this does not allow for precise estimation of a given stimulus. We use visual analog scale (VAS) scores of 10 cm or ordinal scales (e.g., pain ordinal scale) during measurement pauses. Scoring for single stimuli should be enabled by presenting scores during the fMRI experiment. Here, various graphical solutions are used for the evaluation process, e.g., the Self-Assessment Manikin (Bradley and Lang, 1994), asking for two dimensions of emotional rating: valence and arousal. During fMRI measurement, participants score the stimuli using a keypad with at least two choices, navigating up and down the scale, with a start point at the middle. As mentioned, scoring each stimulus during the fMRI measurement adds extensive measuring time.

### Statistical issues in longitudinal fMRI studies

Skup, 2010, already concluded that “the advantage of a longitudinal fMRI approach is that it provides the best possible power for identification of time-related changes because multiple within-subject observations are collected, as long as the variability between subjects is much greater than the variability between sessions for a particular subject.” Another way to address performance differences between participant groups in cross-sectional or longitudinal studies is to include performance in fMRI data analyses. Strother et al., 2004 reported an increase in the power and reproducibility of fMRI analyses when performance parameters were included in first-level analyses. Regarding statistical power, using Bayesian analysis increases power in studies involving participants with performance impairments (Windel et al., 2015). When participants were asked to perform at maximal frequency, a U-shaped fMRI activation was observed both in a longitudinal (Mejia et al., 2022) and a cross-sectional group-comparison design (Loibl et al., 2011). An increase and spreading of fMRI activation during moderate performance is followed by a decrease of fMRI activation in a state of decompensation. In any case, a tight control of performance during fMRI is needed.

### Functional connectivity and the impact of motor performance

Studies on task-related connectivity changes due to motor performance differences are rare. Lin et al. (2009) assessed the relationship between frequency and motor network connectivity. They reported that activity in the lateralized corticocerebellar network shows frequency rate dependence during thumb flexions. The connection between SMA and M1 was also modulated by frequency, but differently for left- and right-hand movements. Another study investigated the effect of three different frequency levels (0.75 Hz, 1.5 Hz, and 3 Hz) using dynamic causal modeling (DCM; Pool et al., 2014). They showed that with higher frequency levels, premotor areas and the cerebellum exerted stronger driving influences on the primary motor cortex. Recently, group comparisons using DCM have been enhanced through the integration of parametric empirical Bayesian procedures, which enable the direct assessment of random effects on connection strengths between participants (Friston et al., 2016).

### Performance control during different modalities of experimental stimulation

**Figure 4:**
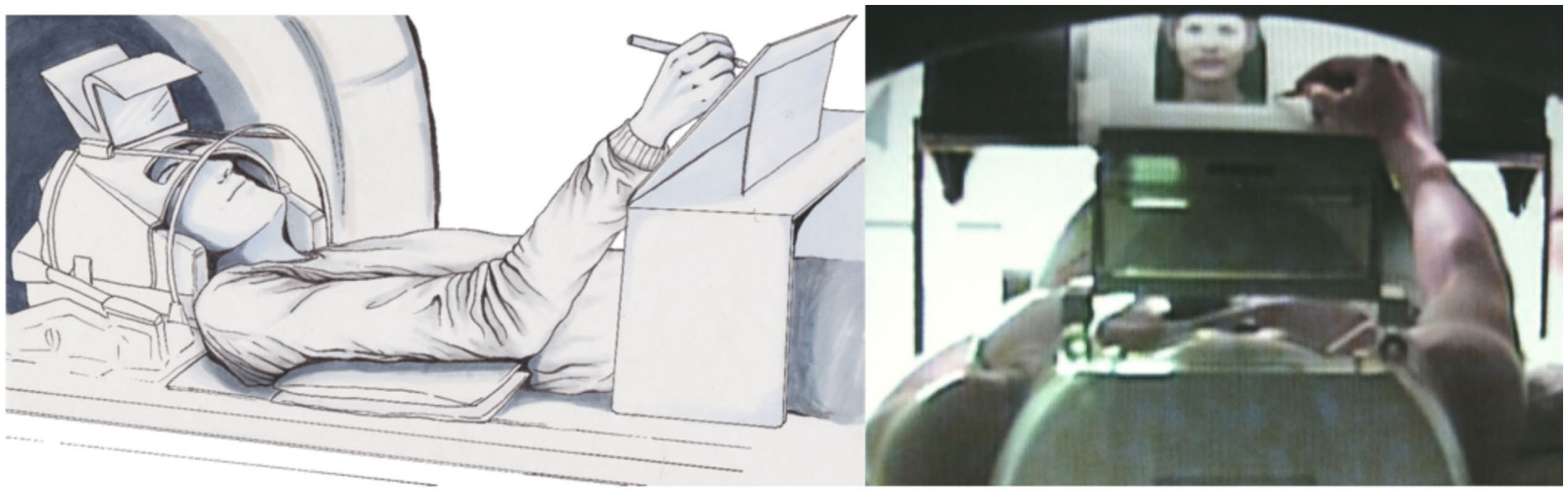
Position of the participant using a writing desk (left: drawing by Ulrike Horn) and a backward view through the scanner hole of the participant scribbling a portrait of a photo (right; Schaer et al., 2012).

A simple pencil and writing board solution can sometimes offer more possibilities than using tablets in the scanner since tablets do not provide feedback in the scanner environment; however, special developments, such as those reported by Reitz et al. (2013), should also be considered. With a simple writing desk, a scanner-suited pencil, and a conventional doubled mirror system (please use a scanner bore as large as possible; see Figure 4A) the complete experiment can be conducted; however, assistance from a person in the scanner room is needed for changing papers on the board (Shah et al., 2013; Erhard et al., 2014). Participants´ performance can then be evaluated offline based on the produced text.

An fMRI-suited tablet [for an overview, see Lin et al. (2021)] can be used to evaluate the preciseness of motor performance. However, since commercially available tablets do not allow to provide feedback on the written text to participants in a scanner environment, we do not use tablets in our studies testing real-life writing or drawing. The main advantage of tablets when compared to paper-and-pencil tasks is the better documentation of the spatial preciseness of participants’ performance.

For the sound domain, fMRI-compatible microphones are commercially distributed, which can record, document, and offline evaluate sounds produced during scanning. For certain tasks, scanner sound might interfere with accurate data evaluation. This can be solved by using software that eliminates scanner sound frequencies or helps in decoding or quantifying auditory responses (e.g., speech recognition software). We use auditory response analyses when test performance is reported verbally (e.g., Grothe et al., 2020) or when identifying laughter intensity in response to tickling (Wattendorf et al., 2019). However, since most sound evaluation processes do not allow for automatized offline analysis, they are usually quite time-consuming. If the scanner sound interferes with a precise analysis of the quality of a produced sound, we apply the sparse sampling technique, enabling scanning brakes of several seconds (we used 3-s measuring brakes) to perform the task (singing a line of an aria; Kleber et al., 2007, 2010). Because of the BOLD delay, the next scan documents the neural response during singing. This method also helps in measuring neurophysiology without scanner artifacts during specific time periods. However, sparse sampling has several disadvantages, such as necessary modifications for fMRI data analyses and lower habituation effects to an optimally constant scanner sound, which might decrease participant compliance.

**Figure 5:**
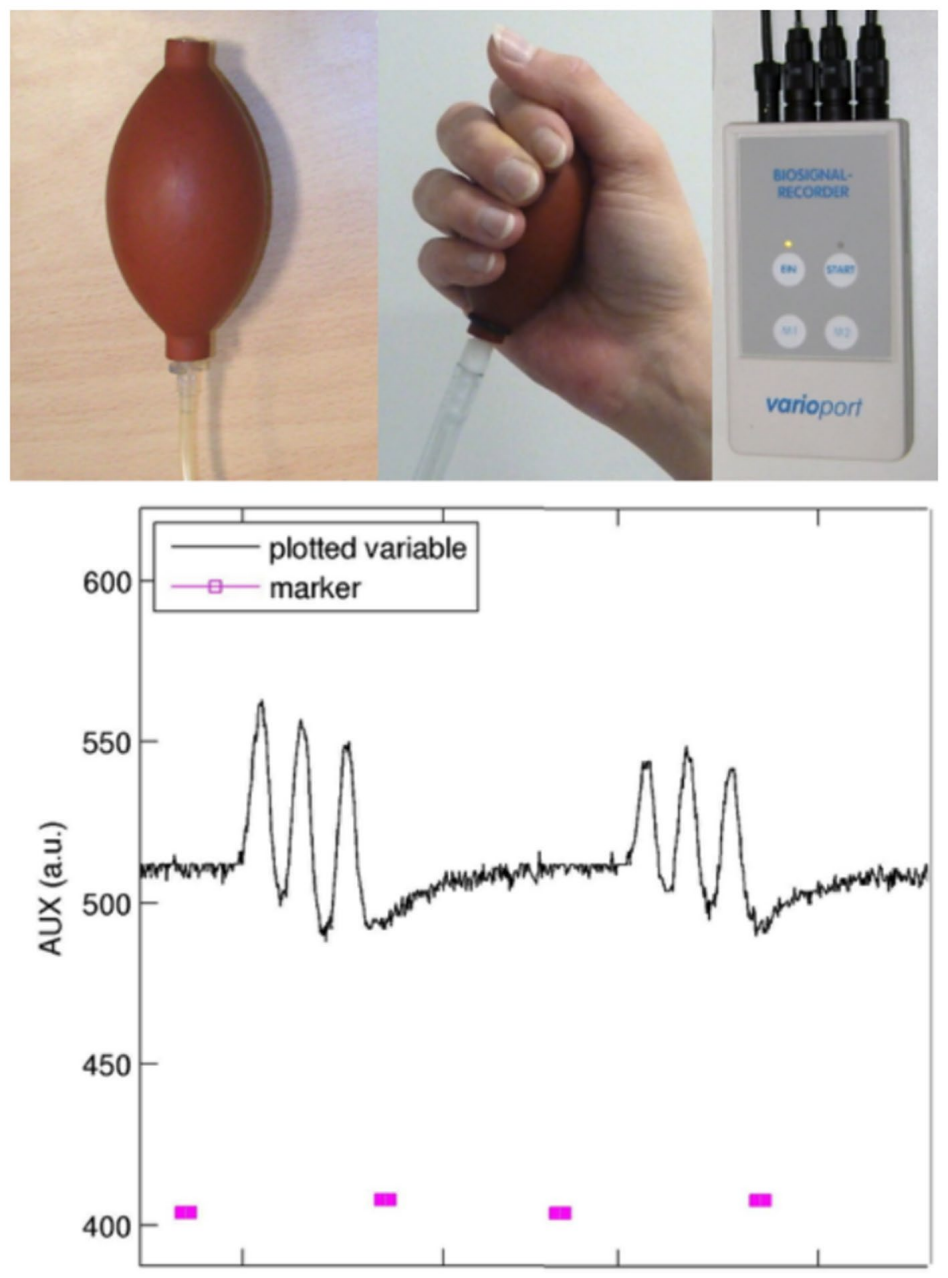
Device for pressure recordings: this device consists of a balloon equipped with a ventil (inflated without losing pressure) and a tube for transducing the air pressure into an electric signal transformer (not shown). The biosignal recorder (e.g., Varioport; BiSigma, Freiburg, Germany) transforms the electrical signal into a light signal, which is then conducted through a Faraday cage to avoid artifacts in MR images. The plot shows on/off markers (pink, bottom) provided to the participant and the recordings of pneumatic changes over time (black top line). Here, repetitive grip movements at a frequency of 1 Hz were performed, with visual feedback given to the participant.

As mentioned in the Introduction, the interpretation of functional representation in sensorimotor performance urgently calls for precise documentation of velocity, force, and preciseness. Therefore, careful performance monitoring in the sensorimotor domain is needed in almost every fMRI center. A very simple solution (see Figure 5) is to build a pressure device with the following key components: a cushion or ball (equipped with a ventil for inserting air and a tube for transmitting pressure) that is fitted to the body part that has to be measured (ball: hand pressure; respiration cushion: swallowing movements; and tube: occlusal movements). The air pressure changes are transformed into electrical signals, which are sent to a biosignal recorder (for instance the varioport system distributed by BiSigma; https://bisigma.de/de/products/amplifier/). The recorder transforms the electric signals into light signals that can be transmitted out of the scanner room and stored for documentation and further evaluation. Different software tools are available for further data processing and monitoring. To use for longitudinal or cross-sectional studies, the complete device should be validated with respect to measurement reliability.

### Specific needs for longitudinal studies in neurorehabilitation

A typical neuroscientific sensorimotor training study involves active repetitive motor training for 20 min, which has been shown to significantly modify the primary motor representation in the amplitude and size of the representation area (Classen et al., 1998). If the task is a single movement in a rigidly presented form (given start, given range, force, and timing), it is perfectly suited for investigating pre–post effects of training [see Lotze et al. (2003)]. However, for patient studies, a rigid performance design is often not possible as they show differential impairments in performance. Therefore, performance has to be individually balanced between the pre- and post- measurements. For balancing performance between measurements in a hand grip task, frequency and grip force are controlled for. Compliance in performing frequency can be controlled by the number of peaks within a block. More difficult is the amplitude adjustment, which can be balanced by absolute measures or by balancing the demand necessary to fulfill the task (Ward et al., 2003). Since impairments vary between patients, we adjust the amplitude by repeatedly measuring maximal force and then train 33% of maximal force pressure by visual feedback. This can be trained offline and then performed without feedback during fMRI or by providing visual feedback during fMRI (Horn et al., 2016). The latter is quite demanding since it involves additional visual, cognitive, and permanent online movement correction capacities. When performing without feedback, the investigator controls online for compliance and starts storing for documentation and offline statistical comparisons. Comparisons for the effect of time (pre, post) or participant groups (e.g., patients, healthy controls) are provided in the final publication.

**Figure 6:**
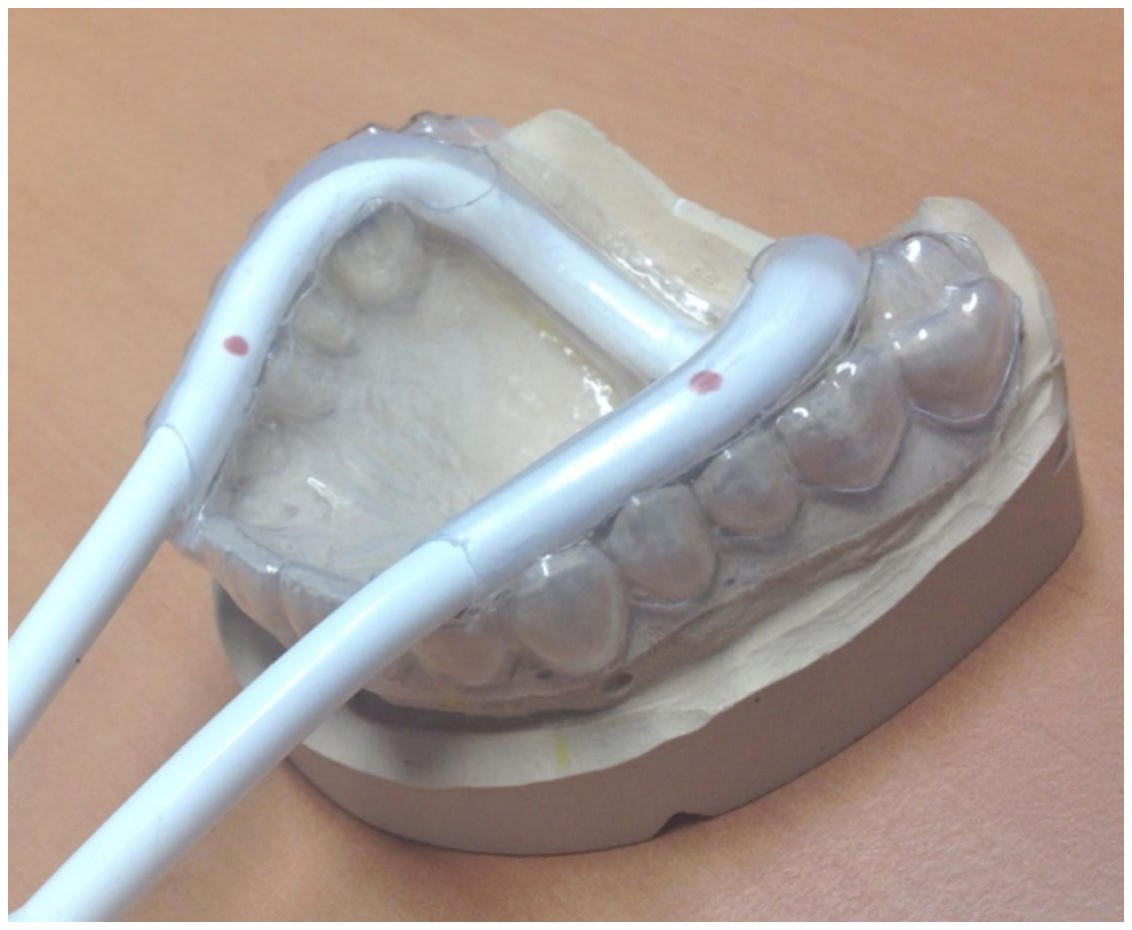
Individually adapted mandibular splint with an integrated pressure tube, coupled with a biosignal recorder for documenting and adjusting occlusal movements. For longitudinal studies, approximately 30% of maximal force can be applied in a block design for measuring about 10 repetitive movements in a row for 4–6 times, alternating with rest [see Klepzig et al. (2024)]. Since pain intensity varies significantly throughout the intervention, the possibility that less pain (during the post-measurement) is associated with a (A) higher frequency, (B) a higher occlusal force, and (C) better compliance has to be documented and excluded. Overall, three different cohorts have now been measured with occlusal performance control under continuous modification and optimization of the device (Lickteig et al., 2013; Ernst et al., 2020; Klepzig et al., 2024).

The biosignal recording device controlling for onset, frequency, and force can be used for different purposes, such as measuring swallowing (see Figure 4) with a respiration cushion affixed around the throat at the height of the cartilage (Mihai et al., 2014, 2016); however, it can also be integrated into a mandibular splint, which has to be adjusted individually. An example for this devise is shown in Figure 6. We use this modified performance documentation for studies on patients with temporomandibular dysfunction to investigate performance in repetitive mandibular occlusal movements (e.g., Klepzig et al., 2024). Other kinematic or EMG measurements are performed offline in a separate session within a given time period around the fMRI experiment (e.g., Lickteig et al., 2013).

### Designs with less demanding performance control

Especially for the acute to the subacute stage of a disease, a paradigm involving active sensorimotor performance is often not possible. This becomes true for more severely impaired patients, such as for patients recovering from a stroke (acute phase: Lindow et al., 2016) or patients with chronic pain (subacute complex regional pain syndrome (CRPS): Gustin et al., 2010). For patients with neurodegenerative diseases (e.g., Mejia et al., 2022), the problem is inverted since cognitive decline progresses over time. For language tasks in patients with aphasia following a stroke, a reading task that compares words against nonsense words is a practical solution to avoid impairments related to decreased language production performance in the acute stage (Saur et al., 2006). For movement tasks, a presentation of pictures or videos of others performing sports has been used for differentiating between obese and non-obese children’s processing of sport-related activities (Davids et al., 2010). Co-activation of the dopamine system during the observation of the sports pictures was shown to be less in children with obesity. This dopamine system co-activation was related to treatment response (less weight increase) following intervention (Kinder et al., 2014). For the motor domain, simple switching from testing active voluntary motor performance to a passively splint-driven performance is not purposeful, although this might solve the problem of achieving a comparable performance during the pre- and post-periods. Voluntary active movements differ substantially in neural representation, training gain, and attention during the task compared to splint-guided passive movements (Lotze et al., 2003). Indeed, fMRI of passive movement performance in an fMRI paradigm on patients with an upper limb impairment following a stroke did not detect any between-group effects (patients with subacute stroke vs. HCs) and did not detect an increase in ventral premotor cortex activation during the post measurement, unlike what was observed during active fist clenching movements by us (Horn et al., 2016) and others (for TMS: Fridman et al., 2004; for real-time fMRI: Sitaram et al., 2012). It might be possible to apply movement observation designs that present videos of active motor performance coupled with a cognitive task (Ertelt et al., 2007). This is already a step in the direction of testing cognitive processing instead of pure movement execution. For the motor domain, one possibility would be to use cognitive tasks, including mental processes that are also used for active motor conditions. For instance, the mental rotation of hands has been termed as implicit motor imagery, which involves a number of regions that are also activated during active motor performance, such as the premotor cortex, the SMA, and parietal areas (Berneiser et al., 2018). In addition, task difficulty should be balanced between the pre- and post-measurements and at both time points, different stimuli should be presented to avoid habituation. It is often useful to produce your own stimulus set optimized for the study’s aim (Walz et al., 2013). This can then be applied in patient studies using cross-sectional (Kohler et al., 2019) or longitudinal (Strauss et al., 2021) designs.

Another possibility might be to switch from the motor domain to the somatosensory domain. However, methodological issues have hindered precise somatosensory mapping with fMRI for decades. New procedures concerning imaging (high resolution, low artifacts) and stimulus presentation (piezotactile vs. air-puff driven stimulation; Schweisfurth et al., 2015), coupled with cognitive tasks to avoid habituation, and methods for high-resolution data evaluation procedures have enabled comparable results for non-invasive human somatosensory mapping (Härtner et al., 2021; Strauss et al., 2021), as previously reported in invasive research on monkeys (Kaas et al., 1979).

## Conclusion

This narrative review described the possibilities to control for performance during different activation fMRI paradigms. Including performance control is essential for almost all activation fMRI paradigms. In addition, several examples were provided for measuring and documenting performance control in the fMRI environment. It might become clear that no active motor fMRI measurement should be performed without controlling for performance. This is especially true for longitudinal protocols in neurorehabilitation, when balancing performance between pre- and post-measurements is a prerequisite for interpreting any differences in fMRI effects over time. However, even in cognitive tasks, testing of performance is needed, which has to be integrated into the measurement protocol. It is challenging to switch from motor performance testing to a more cognitive domain testing implicit motor imagery (mental rotation of hands) or movement observation. For all these tasks, a careful balance of stimulus material between pre- and post-measurements for mental effort and novelty is needed.

After applying active fMRI protocols in neurorehabilitation for three decades, it is quite astonishing that this method has not yet achieved a relevant role in obtaining biomarkers for planning individualized neurorehabilitative interventions. This is mainly due to non-standardized procedures, including little knowledge about controlling participants’ performance during imaging. If these procedures were standardized across research facilities, both the reproducibility of our method and the availability of relevant data in the field would significantly improve. Clear testing of effect sizes through pilot studies, standardized procedures for measurement and data evaluation, including statistical testing, and the definition of each of these aspects in a registered trial are essential for improving the performance of fMRI in neurorehabilitation.
